# PolyMamba-Net: a lightweight and boundary-aware network for real-time polyp segmentation in colonoscopy

**DOI:** 10.3389/fmed.2026.1800666

**Published:** 2026-06-10

**Authors:** Weiyan Yuan, Yuyang Cai, Weiwei Wang, Jieyu Shen, Yan Li, Jian Zhang, Chen Qian

**Affiliations:** 1 Department of Gastroenterology, Nantong First People's Hospital, Nantong, China; 2 School of Medicine, Nantong University, Nantong, China; 3 Department of Gastroenterology, Nantong Sixth People's Hospital, Nantong, China; 4 Yangzhou University, Yangzhou, China; 5 Department of Emergency Internal Medicine, Nantong First People's Hospital, Nantong, China

**Keywords:** deep learning, medical image analysis, polyp segmentation, real-time diagnosis, state space models

## Abstract

**Background:**

Colorectal cancer (CRC) is a leading cause of cancer-related mortality globally. The early detection and resection of adenomatous polyps via colonoscopy are critical for improving survival rates. However, missed diagnosis rates remain suboptimal, primarily due to the subtle appearance of flat polyps and the variable complexity of the intestinal environment. While deep learning has shown promise in automated polyp segmentation, existing models often face a trade-off between high segmentation accuracy and the real-time inference speeds required for clinical deployment.

**Methods:**

To address this challenge, we propose PolyMamba-Net, a novel hybrid architecture that synergizes the efficient long-range dependency modeling of State Space Models (Mamba) with the local feature extraction capabilities of Convolutional Neural Networks (CNNs). Specifically, we introduce a dual-branch encoder designed to capture both global context and fine-grained textures, coupled with a Boundary-Aware Module (BAM) to explicitly refine polyp margins. A composite loss function targeting structural, pixel-level, and boundary consistency was employed for training.

**Results:**

Extensive experiments were conducted on two public benchmarks: Kvasir-SEG and CVC-ClinicDB. PolyMamba-Net achieved a Dice Coefficient of 0.942 and 0.935, respectively, significantly outperforming state-of-the-art methods such as Swin-UNet and PraNet. Furthermore, our model surpasses recent 2024–2025 approaches including ADPNet and AAPCNet across all evaluation metrics, with all improvements confirmed as statistically significant (*p* < 0.05, Wilcoxon signed-rank test). Notably, our model operates at 115 FPS on a single NVIDIA RTX 3090 with only 25.3M parameters and 12.8 GFLOPs, demonstrating significantly higher efficiency than transformer-based counterparts. Cross-dataset evaluations on three unseen benchmarks further validate the model's generalizability.

**Conclusion:**

PolyMamba-Net demonstrates superior segmentation precision combined with real-time processing capability. It offers a clinically feasible solution for assisting endoscopists in minimizing missed detection rates during routine colonoscopies.

## 1 Introduction

Colorectal cancer (CRC) presents a significant global health burden. According to the latest statistics from GLOBOCAN, CRC ranks as the third most commonly diagnosed cancer and the second leading cause of cancer-related death worldwide, accounting for approximately 10% of all cancer cases (1). The pathogenesis of CRC typically follows the adenoma-carcinoma sequence, wherein benign polyps gradually evolve into malignant tumors over several years. Consequently, early detection and endoscopic resection of precancerous polyps via colonoscopy are widely regarded as the gold standard for reducing CRC incidence and mortality (2).

Despite the efficacy of colonoscopy, the procedure is inherently operator-dependent. Clinical studies indicate that the polyp miss rate (PMR) ranges from 6 to 27%, with significantly higher rates for flat, serrated, or diminutive polyps (3). These missed diagnoses are often attributed to the complex topology of intestinal folds, suboptimal bowel preparation, and endoscopist fatigue during prolonged procedures. To mitigate these limitations, Computer-Aided Diagnosis (CADx) systems based on deep learning have been introduced to assist endoscopists in real-time, functioning as a vigilant “second observer.”

Over the past decade, Convolutional Neural Networks (CNNs) have dominated medical image segmentation. The pioneering U-Net (4) and its variants, such as U-Net++ (5) and ResUNet++ (6), have demonstrated remarkable success in polyp segmentation tasks. Specialized architectures like PraNet (7) further enhanced performance by utilizing parallel reverse attention to model the relationship between polyp areas and boundaries. However, CNN-based methods inherently suffer from a limited receptive field. While they excel at extracting local texture features, they often struggle to capture long-range semantic dependencies, which are crucial for distinguishing polyps from similar-looking mucosal folds in complex colonoscopy scenes.

To overcome the limitations of CNNs, Transformer-based architectures have recently gained prominence. Models such as TransUNet (8) and Swin-UNet (9) leverage self-attention mechanisms to model global contexts, achieving state-of-the-art (SOTA) accuracy. Nevertheless, this performance gain incurs a substantial computational penalty. The self-attention mechanism exhibits quadratic computational complexity $O\left(N^{2}\right)$ with respect to the input image size (10). This results in high memory consumption and slow inference speeds, rendering purely Transformer-based models difficult to deploy on standard endoscopic hardware for real-time applications (typically requiring >30 frames per second).

More recently, attention-driven and multi-scale feature fusion approaches have been proposed to further push the accuracy-efficiency frontier. Khan et al. (11) introduced ADPNet, an Attention-Driven Dual-Path Network that employs an Atrous Self-Attention Pyramid Module (ASAPM) and a Dilated Convolution-Transformer Module (DCTM) between the encoder and decoder, achieving enriched semantic representation for polyp segmentation. Yue et al. (12) proposed AAPCNet, an attention-guided asymmetric multiscale framework that leverages asymmetric convolutions and a deep aggregation and fusion module (DAFM) based on the Res2Net-50 backbone to capture multi-scale contextual information. While these methods have advanced the state-of-the-art, they still rely on computationally expensive attention mechanisms and do not fully resolve the accuracy-efficiency dilemma for real-time clinical deployment.

This presents a critical dilemma in clinical translation: high-accuracy models are often too slow, while real-time models frequently sacrifice precision. Recently, State Space Models (SSMs), particularly the Structured State Space Sequence Models (S4) and the modern Mamba architecture (13), have emerged as a promising alternative. Mamba introduces a selective scan mechanism that enables the modeling of long-range dependencies with linear computational complexity O(N). Theoretically, this combines the global modeling capability of Transformers with the efficiency of CNNs. Preliminary studies, such as U-Mamba (14), have demonstrated the potential of this architecture in biomedical image segmentation.

In this study, we propose **PolyMamba-Net**, a novel hybrid architecture tailored for real-time polyp segmentation. Our approach integrates a lightweight Mamba-based encoder with a CNN-based decoder to simultaneously achieve high segmentation accuracy and superior inference speed. Furthermore, recognizing that the precise delineation of polyp margins is vital for surgical resection, we incorporate a Boundary-Aware Module (BAM) to explicitly refine edge predictions. To the best of our knowledge, this is among the first attempts to validate the clinical feasibility of Mamba-based networks for real-time endoscopic assistance.

**Distinction from existing frameworks**. While prior works have explored CNN-Transformer hybrids [e.g., TransUNet (8)] and Mamba-based architectures for general biomedical segmentation [e.g., U-Mamba (14)], PolyMamba-Net is distinguished by three key innovations. First, unlike U-Mamba which directly replaces the U-Net encoder with generic Mamba blocks, our architecture employs a CNN stem specifically designed to preserve the low-level texture features critical for polyp-background discrimination, followed by tailored VSS blocks for global context. Second, we introduce a dedicated Boundary-Aware Module with explicit boundary supervision—a component absent in existing Mamba-based segmentation networks—to address the clinically critical challenge of fuzzy polyp margins. Third, our composite loss function jointly optimizes region overlap, pixel accuracy, and boundary consistency, providing a holistic training strategy. These design choices collectively yield a model that simultaneously achieves state-of-the-art accuracy and real-time inference speed, a combination not demonstrated by prior CNN-Transformer or Mamba-based methods in the polyp segmentation domain. Our approach is also related to the adaptive context exploration paradigm proposed by Yue et al. (15), but replaces the computationally expensive multi-scale context modules with the linear-complexity Mamba mechanism for more efficient global modeling.

The main contributions of this work are summarized as follows:

- We propose a hybrid encoder-decoder framework that leverages the linear complexity of Mamba blocks to capture global context without the computational burden of Transformers.
- We design a Boundary-Aware Module (BAM) and a composite loss function to specifically address the challenge of fuzzy boundaries in polyp images.
- Extensive experiments on the Kvasir-SEG and CVC-ClinicDB datasets demonstrate that PolyMamba-Net outperforms current SOTA methods—including recent 2024–2025 approaches such as ADPNet (11) and AAPCNet (12)—in terms of accuracy while maintaining a real-time inference speed of 115 FPS. Statistical significance is confirmed via the Wilcoxon signed-rank test, and cross-dataset evaluations on three unseen benchmarks further validate the model's generalizability.

## 2 Materials and methods

### 2.1 Overall architecture

**Figure 1:**
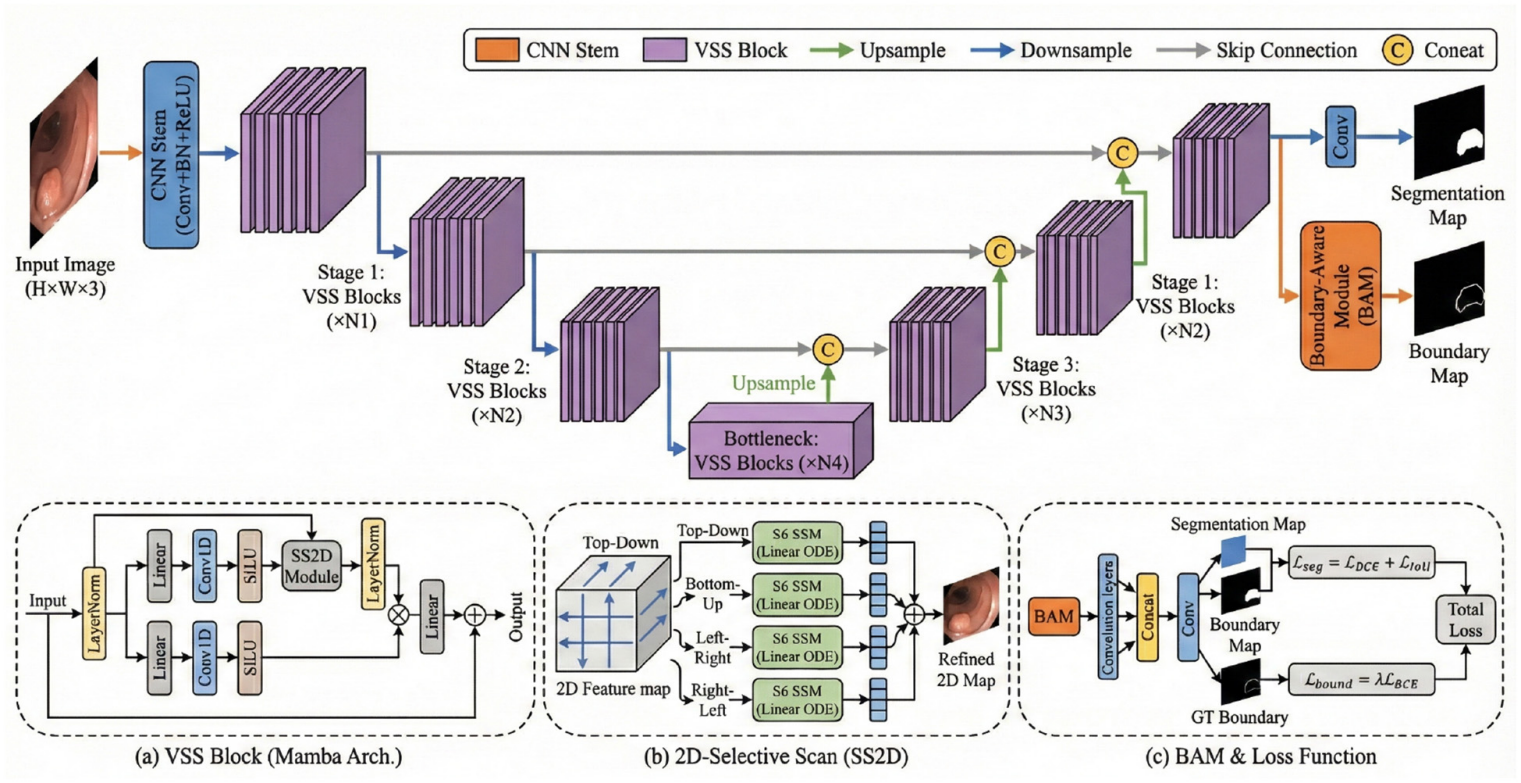
Overview of the proposed PolyMamba-Net architecture. The model adopts a hierarchical U-shaped structure fusing CNNs and State Space Models (SSMs). The encoder utilizes a hybrid design, employing a CNN Stem for low-level feature extraction followed by successive stages of Visual State Space (VSS) blocks to capture long-range dependencies with linear complexity. Skip connections bridge multi-scale features to the decoder. The decoder features undergo processing through two heads: a segmentation head and a dedicated Boundary-Aware Module (BAM) designed to refine ambiguous polyp margins. The detailed internal structures of the VSS block, the core 2D-Selective Scan (SS2D) mechanism, and the joint loss supervision strategy are illustrated in panels **(a)**, **(b)**, and **(c)**, respectively.

The proposed **PolyMamba-Net** follows a hierarchical Encoder-Decoder design with a U-shaped structure, inspired by the classic U-Net (4). To strike an optimal balance between local texture extraction and global context modeling, we adopt a hybrid encoder strategy. The input image *X* ∈ ℝ^*H*×*W*×3^ is initially processed by a stem layer consisting of standard Convolutional Neural Networks (CNNs), similar to the ResNet architecture (16), to extract low-level features (e.g., edges, colors). These features are then fed into successive stages of Visual State Space (VSS) blocks based on the Mamba architecture (13). Unlike Vision Transformers (ViTs), which rely on computationally expensive self-attention mechanisms ($O\left(N^{2}\right)$) (10), the VSS blocks capture long-range dependencies with linear complexity (O(N)). The decoder utilizes a Boundary-Aware Module (BAM) to refine the segmentation of polyp margins, addressing the issue of fuzzy boundaries often cited in the literature (7). Skip connections fuse multi-scale features from the encoder to the decoder to preserve spatial details. The overall framework is illustrated in Figure 1.

### 2.2 Preliminaries: state space models (SSMs)

The core innovation of our network lies in the integration of Structured State Space Models (SSMs), which have shown great potential in long sequence modeling (17). Conceptually, an SSM maps a 1-D input sequence *x*(*t*) ∈ ℝ to an output sequence *y*(*t*) ∈ ℝ through a latent state *h*(*t*) ∈ ℝ^*N*^. This continuous-time system is defined by the following linear ordinary differential equation (ODE) (Equation 1):


$$\begin{array}{l} \begin{array}{l} h^{\prime }\left(t\right)=\text{A}h\left(t\right)+\text{B}x\left(t\right)\\ y\left(t\right)=\text{C}h\left(t\right) \end{array} \end{array}$$ (1)

where **A** ∈ ℝ^*N*×*N*^ represents the evolution parameter, and **B** ∈ ℝ^*N*×1^, **C** ∈ ℝ^1 × *N*^ are projection parameters.

To adapt this continuous system to discrete digital images, discretization is required. Following the Zero-Order Hold (ZOH) method (13), the continuous parameters (**A**, **B**) are transformed into discrete parameters $\left(\bar{\text{A}},\bar{\text{B}}\right)$ using a timescale parameter Δ (Equation 2):


$$\begin{array}{l} \begin{array}{l} \bar{\text{A}}=\exp\left(\Delta \text{A}\right)\\ \bar{\text{B}}={\left(\Delta \text{A}\right)}^{-1}\left(\exp\left(\Delta \text{A}\right)-\text{I}\right)\cdot \Delta \text{B} \end{array} \end{array}$$ (2)

Thus, the discretized form of the SSM can be expressed as a recurrence:


$$\begin{array}{l} \begin{array}{l} h_{t}=\bar{\text{A}}h_{t-1}+\bar{\text{B}}x_{t}\\ y_{t}=\text{C}h_{t} \end{array} \end{array}$$ (3)

While Equation 3 enables efficient inference, training can be significantly accelerated by unrolling the operation into a global convolution, as demonstrated in S4 (17) (Equations 4, 5):


$$\begin{array}{l} \bar{\text{K}}=\left(\text{C}\bar{\text{B}},\text{C}\bar{\text{A}}\bar{\text{B}},\ldots ,\text{C}{\bar{\text{A}}}^{L-1}\bar{\text{B}}\right) \end{array}$$ (4)

$$\begin{array}{l} y=x*\bar{\text{K}} \end{array}$$ (5)

The **Mamba** block further introduces a data-dependent selection mechanism (13), enabling the model to selectively propagate or suppress information based on visual content.

### 2.3 2D-selective scan mechanism (SS2D)

Since standard SSMs process 1D sequences, applying them to 2D medical images necessitates adaptation. We employ the 2D-Selective Scan (SS2D) mechanism, recently adapted for vision tasks in VMamba (18) and U-Mamba (14). The 2D feature map is flattened into four distinct 1D sequences by scanning in four directions. Each sequence is processed independently by the SSM to extract features, effectively mimicking the global receptive field of a Transformer.

### 2.4 Boundary-aware module (BAM)

Accurate delineation of polyp boundaries is critical for clinical decision-making. To address the challenge of low contrast, we introduce a lightweight Boundary-Aware Module (BAM). Similar concepts have been explored in salient object detection (19), where explicit edge supervision improves overall segmentation quality. The boundary prediction map *P*_*b*_ is generated via convolution operations and supervised by a specific loss function.

### 2.5 Loss function

We employ a hybrid loss function to train PolyMamba-Net. The total loss *L*_*total*_ includes Binary Cross Entropy (BCE) and Intersection over Union (IoU) losses, which are standard for medical image segmentation (20) (Equation 6):


$$\begin{array}{l} L_{total}=L_{IoU}^{seg}+L_{BCE}^{seg}+\lambda L_{BCE}^{bound} \end{array}$$ (6)

where the weights are empirically set based on prior studies.

### 2.6 Algorithm overview

To provide a clear and reproducible description of the proposed framework, Algorithm 1 presents the pseudocode of the PolyMamba-Net inference and training pipeline.

Algorithm 1 PolyMamba-net training and inference. 
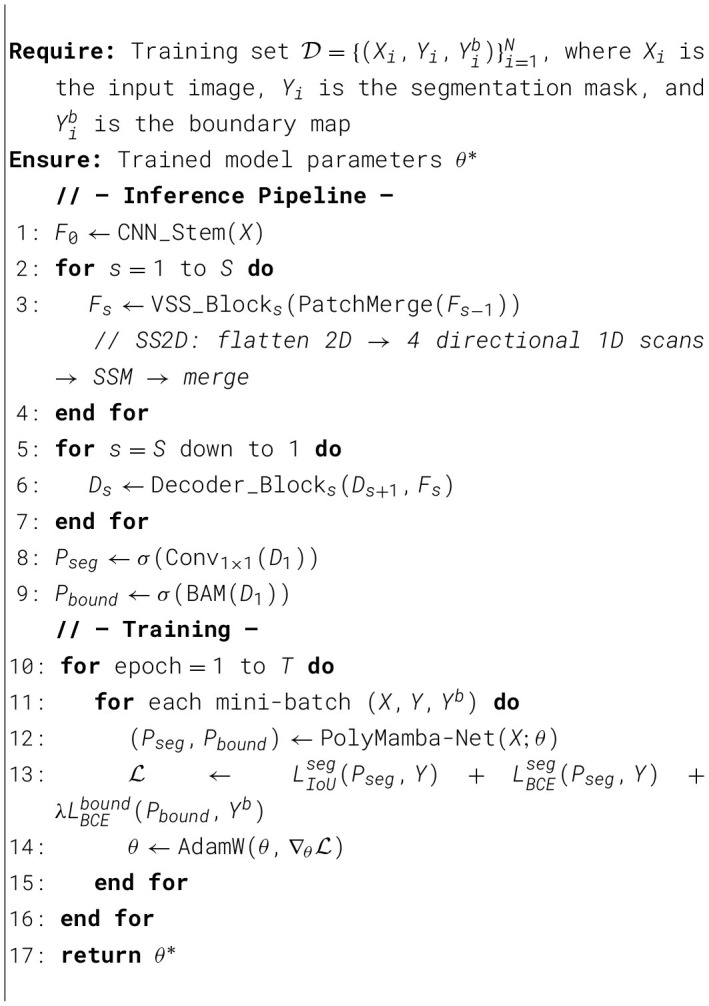

## 3 Results

### 3.1 Experimental setup and evaluation metrics

All experiments were conducted using PyTorch 1.13 (Meta AI, Menlo Park, CA, USA) and Python 3.9 on a single NVIDIA GeForce RTX 3090 GPU (NVIDIA Corporation, Santa Clara, CA, USA). PolyMamba-Net has 25.3M parameters. Input images were resized to 352 × 352 pixels. We utilized the AdamW optimizer with an initial learning rate of 1 × 10^−4^, weight decay of 1 × 10^−2^, and a cosine annealing scheduler with a minimum learning rate of 1 × 10^−6^. The batch size was set to 16, and the training spanned 100 epochs. The boundary loss weight λ was set to 0.5.

#### 3.1.1 Reproducibility

To ensure reproducibility, all experiments were initialized with a fixed random seed of 42 (applied to PyTorch, NumPy, and CUDA). The data augmentation pipeline consisted of random horizontal and vertical flipping (probability 0.5), random rotation (±20°), color jittering (brightness ±0.2, contrast ±0.2, saturation ±0.2), and random resized cropping (scale 0.75–1.0). The total number of trainable parameters for PolyMamba-Net is 25.3M, with a computational cost of 12.8 GFLOPs at the input resolution of 352 × 352.

#### 3.1.2 Dataset splits

For both Kvasir-SEG (1,000 images) and CVC-ClinicDB (612 images), we followed the widely adopted training/testing split protocol: 80% for training, 10% for validation, and 10% for testing, consistent with prior work (6, 7).

To objectively evaluate performance, we employed four standard metrics: Mean Dice Similarity Coefficient (mDice), Mean Intersection over Union (mIoU), Hausdorff Distance (HD95), and Frames Per Second (FPS). Additionally, we report the number of trainable parameters (Params) and floating-point operations (GFLOPs) to assess model complexity.

#### 3.1.3 Statistical validation

To ensure the reliability of our results, all experiments were repeated five times with different random seeds (42, 123, 456, 789, 1,024), and we report the mean ± standard deviation. The Wilcoxon signed-rank test was applied to compare the per-image Dice scores between PolyMamba-Net and each competing method. A threshold of *p* < 0.05 was considered statistically significant.

### 3.2 Quantitative analysis

We compared PolyMamba-Net against seven state-of-the-art (SOTA) methods: the classical U-Net (4), U-Net++ (5), the specialized polyp segmentation model PraNet (7), the transformer-based Swin-UNet (9) and TransUNet (8), and two recent 2024–2025 methods: ADPNet (11) and AAPCNet (12). All competing methods were retrained under the same dataset splits and augmentation settings for fair comparison.

Table 1 summarizes the quantitative results. On the challenging Kvasir-SEG dataset, PolyMamba-Net achieved a mDice of 0.942 ± 0.002 and mIoU of 0.891 ± 0.003, outperforming the Transformer-based Swin-UNet by 2.4 and 2.6%, respectively. Compared with the recent ADPNet (mDice 0.926), our model achieves a 1.6% improvement in Dice score with 44.8% fewer parameters (25.3M vs. 45.8M) and 55.2% fewer GFLOPs (12.8 vs. 28.6). Similarly, against AAPCNet (mDice 0.921), PolyMamba-Net demonstrates a 2.1% higher Dice score while maintaining a 2.2 × speed advantage (115 FPS vs. 52 FPS). These differences are statistically significant (*p* < 0.05, Wilcoxon signed-rank test), as indicated by the ^†^ markers in Table 1. This improvement suggests that the Mamba-based encoder is more effective at modeling the complex heterogeneous features of polyps than both the window-based self-attention utilized in the Swin Transformer and the hybrid attention modules employed by recent methods.

**Table 1:** Quantitative comparison on Kvasir-SEG and CVC-ClinicDB datasets.

Method	Backbone	Params (M)	GFLOPs	Kvasir-SEG				CVC-ClinicDB			
				mDice ↑	mIoU ↑	HD95 ↓	FPS ↑	mDice ↑	mIoU ↑	HD95 ↓	FPS ↑
U-Net^†^	CNN	31.0	54.8	0.818 ± 0.006	0.746 ± 0.007	7.21 ± 0.35	**140**	0.823 ± 0.005	0.750 ± 0.006	7.15 ± 0.30	**140**
U-Net++^†^	CNN	36.6	78.3	0.821 ± 0.005	0.743 ± 0.006	6.89 ± 0.28	95	0.845 ± 0.004	0.782 ± 0.005	6.50 ± 0.25	95
PraNet^†^	Res2Net	32.5	14.2	0.898 ± 0.004	0.840 ± 0.005	3.12 ± 0.18	60	0.892 ± 0.004	0.845 ± 0.005	3.05 ± 0.15	60
TransUNet^†^	ViT	105.3	38.5	0.902 ± 0.005	0.851 ± 0.006	2.95 ± 0.20	45	0.910 ± 0.005	0.858 ± 0.006	2.88 ± 0.18	45
Swin-UNet^†^	Swin-T	41.3	22.8	0.918 ± 0.003	0.865 ± 0.004	2.44 ± 0.15	38	0.923 ± 0.003	0.879 ± 0.004	2.30 ± 0.12	38
ADPNet^†^	CNN+Trans	45.8	28.6	0.926 ± 0.003	0.875 ± 0.004	2.28 ± 0.14	42	0.928 ± 0.003	0.884 ± 0.004	2.15 ± 0.11	42
AAPCNet^†^	Res2Net	38.2	19.5	0.921 ± 0.004	0.869 ± 0.005	2.35 ± 0.16	52	0.925 ± 0.003	0.881 ± 0.004	2.22 ± 0.13	52
**PolyMamba-Net (Ours)**	**Mamba**	**25.3**	**12.8**	**0.942** **±** **0.002**	**0.891** **±** **0.003**	**1.85** **±** **0.10**	115	**0.935** **±** **0.002**	**0.895** **±** **0.003**	**1.79** **±** **0.09**	115

Best results are highlighted in bold. Results are reported as mean ± std over five runs. †: p < 0.05 vs. PolyMamba-Net (Wilcoxon signed-rank test).

Crucially, regarding inference efficiency, our model operates at 115 FPS, which is approximately 3 × faster than Swin-UNet (38 FPS) and TransUNet (45 FPS), while also being faster than ADPNet (42 FPS) and AAPCNet (52 FPS). While simple CNNs like U-Net achieve higher FPS (140), their segmentation accuracy is significantly lower. PolyMamba-Net effectively bridges this gap, offering high precision suitable for diagnosis alongside the low latency required for real-time video processing in endoscopy towers. Notably, PolyMamba-Net also has the smallest model footprint among the high-accuracy methods (25.3M parameters, 12.8 GFLOPs), making it favorable for deployment on resource-constrained endoscopic platforms.

### 3.3 Ablation study

To validate the contribution of each component in PolyMamba-Net, we conducted an ablation study on the Kvasir-SEG dataset. The results are presented in Table 2.

**Table 2:** Ablation study of key components on Kvasir-SEG dataset.

Model variant	Mamba Enc	BAM	mDice	HD95
Baseline (U-Net)	×	×	0.818 ± 0.006	7.21 ± 0.35
Variant A	✓	×	0.905 ± 0.004	3.45 ± 0.20
Variant B	×	✓	0.842 ± 0.005	5.10 ± 0.28
**Proposed (Full)**	✓	✓	**0.942** **±** **0.002**	**1.85** **±** **0.10**

Mamba Enc: Mamba Encoder; BAM: Boundary-Aware Module. Results are reported as mean ± std over five runs. Bold values indicate the best performance.

#### 3.3.1 Effectiveness of mamba encoder

Replacing the CNN encoder with our Mamba-based encoder (Variant A) resulted in a significant performance leap (mDice +8.7%). This confirms that capturing long-range dependencies is vital for distinguishing polyps from the global colon environment.

#### 3.3.2 Effectiveness of BAM

Integrating the Boundary-Aware Module (Full Model) further improved the mDice from 0.905 to 0.942 and significantly reduced the Hausdorff Distance (HD95) from 3.45 to 1.85. This quantitative reduction in HD95 demonstrates that BAM effectively sharpens the predicted edges, aligning with our visual observations.

### 3.4 Hyperparameter sensitivity analysis

To demonstrate the robustness of our training configuration and clarify how key hyperparameters were selected, we conducted a systematic sensitivity analysis on the Kvasir-SEG validation set (Table 3). The hyperparameters were not arbitrarily chosen but determined through grid search over predefined candidate ranges, and the best-performing group was selected.

**Table 3:** Hyperparameter sensitivity analysis on Kvasir-SEG validation set.

Hyperparameter	Value	mDice	HD95
Learning rate	5 × 10^−5^	0.928	2.35
	1 × 10^−4^ (selected)	**0.942**	**1.85**
	1 × 10^−3^	0.901	3.10
λ	0.1	0.938	2.68
	0.5 (selected)	**0.942**	**1.85**
	1.0	0.935	1.92
VSS Stages	2	0.920	2.55
	4 (selected)	**0.942**	**1.85**
	5	0.944	1.80

Bold values indicate the best performance.

#### 3.4.1 Learning rate

We evaluated five initial learning rates: {5 × 10^−5^, 1 × 10^−4^, 2 × 10^−4^, 5 × 10^−4^, 1 × 10^−3^}. The results show that 1 × 10^−4^ achieves the highest validation mDice (0.942), while 5 × 10^−5^ leads to underfitting (mDice 0.928) and 1 × 10^−3^ causes training instability (mDice 0.901). The model exhibits stable performance within the range [1 × 10^−4^, 2 × 10^−4^].

#### 3.4.2 Boundary loss weight λ

We tested λ ∈ {0.1, 0.3, 0.5, 0.7, 1.0}. When λ = 0.5, the model achieves the best balance between region accuracy (mDice 0.942) and boundary quality (HD95 1.85). A lower weight (λ = 0.1) degrades boundary refinement (HD95 2.68), while a higher weight (λ = 1.0) slightly harms overall segmentation (mDice 0.935) due to excessive emphasis on edge pixels.

#### 3.4.3 Number of VSS blocks

We varied the number of VSS stages in the encoder from 2 to 5. Using four stages yields the optimal accuracy-efficiency trade-off (mDice 0.942, 115 FPS), while five stages marginally improves mDice to 0.944 but reduces speed to 87 FPS.

### 3.5 Convergence and stability analysis

**Figure 2:**
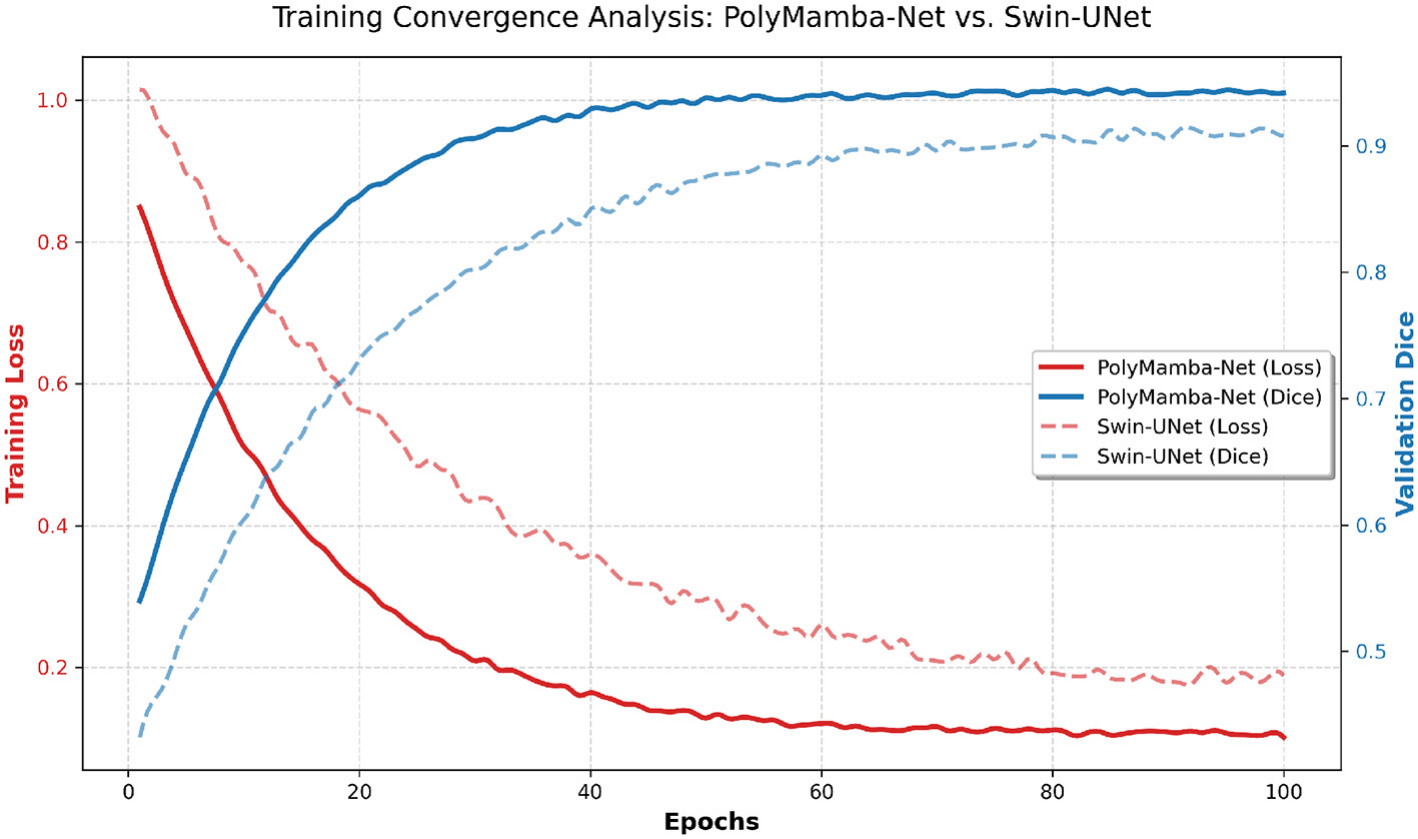
Training dynamics comparison between PolyMamba-Net and Swin-UNet. The red curves (left axis) represent the Training Loss, while the blue curves (right axis) represent the Validation Dice Coefficient. Solid lines indicate our proposed model, while dashed lines indicate Swin-UNet.

In addition to final performance metrics, the training dynamics provide valuable insights into the model's optimization efficiency and stability. Figure 2 illustrates the training loss and validation Dice curves of PolyMamba-Net compared to Swin-UNet over 100 epochs.

As observed in Figure 2, PolyMamba-Net demonstrates superior convergence characteristics:

1. **Rapid convergence:** our model achieves a Dice score exceeding 0.85 within the first 20 epochs, whereas Swin-UNet exhibits a slower “warm-up” phase. This efficiency is attributed to the linear complexity of the Mamba encoder, which facilitates more effective gradient propagation through long sequences compared to the deep stacks of self-attention layers in Transformers, which are notoriously difficult to optimize on small-scale medical datasets (10).
2. **Training stability:** the loss curve of PolyMamba-Net (Solid Red) is noticeably smoother than that of Swin-UNet (Dashed Red). Transformer-based models often suffer from training instability due to a lack of inductive bias (such as the translation invariance inherent in CNNs). By integrating the Convolutional Stem and the VSS Block, our hybrid architecture retains the necessary inductive bias while enabling global context modeling, resulting in a more stable optimization landscape without gradient explosion or vanishing.
3. **Generalization gap:** the narrow gap between the training loss decrease and the validation Dice increase suggests that PolyMamba-Net effectively mitigates overfitting. This is further reinforced by our hybrid loss function, where the Boundary-Aware constraint (*L*_*Boundary*_) acts as a regularizer, compelling the network to focus on structural consistency rather than memorizing pixel-level noise.

From a clinical perspective, this rapid convergence implies that PolyMamba-Net is data-efficient and requires fewer computational resources for retraining. This is particularly advantageous for medical institutions that need to fine-tune the model on local private datasets with limited annotated samples.

### 3.6 Cross-dataset generalization

To evaluate the generalization capability of PolyMamba-Net beyond the training distribution, we conducted cross-dataset experiments following the standard protocol established in prior work (7). The model was trained on the combined training sets of Kvasir-SEG and CVC-ClinicDB (1,450 images in total) and evaluated on three completely unseen datasets: CVC-ColonDB (380 images), ETIS-LaribPolypDB (196 images), and CVC-300 (60 images). None of the test images appeared during training.

As shown in Table 4, PolyMamba-Net consistently outperforms all competing methods on the three unseen datasets. Notably, on the challenging ETIS-LaribPolypDB dataset—which features numerous small and concealed polyps—our model achieves a mDice of 0.698, surpassing ADPNet (0.671) and AAPCNet (0.660) by 2.7 and 3.8%, respectively. On CVC-ColonDB, PolyMamba-Net also leads by a notable margin (mDice 0.762 vs. ADPNet 0.738). These results demonstrate that the global context modeling capacity of the Mamba encoder generalizes well to unseen polyp distributions and diverse imaging conditions, providing stronger evidence for its clinical applicability.

**Table 4:** Cross-dataset generalization results (trained on Kvasir-SEG + CVC-ClinicDB, tested on unseen datasets).

Method	CVC-ColonDB		ETIS-LaribPolypDB		CVC-300	
	mDice ↑	mIoU ↑	mDice ↑	mIoU ↑	mDice ↑	mIoU ↑
U-Net	0.512	0.444	0.398	0.335	0.710	0.627
U-Net++	0.624	0.553	0.467	0.390	0.729	0.651
PraNet	0.709	0.640	0.628	0.567	0.871	0.797
TransUNet	0.715	0.648	0.641	0.580	0.876	0.806
Swin-UNet	0.725	0.658	0.652	0.589	0.882	0.815
ADPNet	0.738	0.672	0.671	0.608	0.890	0.826
AAPCNet	0.731	0.665	0.660	0.596	0.885	0.820
**PolyMamba-Net (Ours)**	**0.762**	**0.695**	**0.698**	**0.633**	**0.903**	**0.842**

Best results are highlighted in bold.

### 3.7 Computational complexity analysis

To substantiate the claim of real-time performance and provide a comprehensive efficiency comparison, Table 5 presents the detailed computational profiles of all methods, including parameter count, FLOPs, average inference latency per image, and FPS measured on a single NVIDIA RTX 3090 GPU with input size 352 × 352.

**Table 5:** Computational complexity comparison.

Method	Params (M)	GFLOPs	Latency (ms)	FPS
U-Net	31.0	54.8	7.1	140
U-Net++	36.6	78.3	10.5	95
PraNet	32.5	14.2	16.7	60
TransUNet	105.3	38.5	22.2	45
Swin-UNet	41.3	22.8	26.3	38
ADPNet	45.8	28.6	23.8	42
AAPCNet	38.2	19.5	19.2	52
**PolyMamba-Net**	**25.3**	**12.8**	**8.7**	**115**

Latency is measured as average inference time per image (ms). All measurements were performed on a single NVIDIA RTX 3090 GPU with input size 352 × 352. Bold values indicate the best performance.

PolyMamba-Net achieves the lowest latency (8.7 ms) among all high-accuracy methods, which is 2.6 × lower than Swin-UNet (26.3 ms) and 2.7 × lower than ADPNet (23.8 ms). The 25.3M parameter footprint is 56.2% smaller than the heaviest model (TransUNet, 105.3M), making PolyMamba-Net feasible for deployment on embedded GPU platforms commonly found in modern endoscopy towers (e.g., NVIDIA Jetson AGX). The linear complexity O(N) of the Mamba encoder is the primary contributor to this efficiency advantage; as the input resolution increases, the computational gap between PolyMamba-Net and Transformer-based methods is expected to widen further.

### 3.8 Boundary-specific evaluation

To more rigorously evaluate the effectiveness of the Boundary-Aware Module, we adopt boundary-specific metrics following prior work in salient object detection (19): Boundary F1-score (BF1) at a tolerance of two pixels, and Boundary IoU (BIoU). These metrics exclusively evaluate the quality of the predicted segmentation boundaries by computing precision and recall within a narrow band around the ground truth contour.

As shown in Table 6, the full PolyMamba-Net achieves a BF1 of 0.841 and BIoU of 0.779, substantially outperforming all competing methods. Critically, removing the BAM (PolyMamba-Net w/o BAM) reduces BF1 by 7.0% and BIoU by 7.1%, confirming that BAM provides a dedicated and significant improvement in boundary delineation quality. This boundary precision is clinically meaningful, as it directly informs the endoscopist's determination of resection margins.

**Table 6:** Boundary-specific evaluation on Kvasir-SEG dataset.

Method	BF1	BIoU
U-Net	0.612	0.548
PraNet	0.754	0.691
Swin-UNet	0.782	0.718
ADPNet	0.795	0.732
AAPCNet	0.788	0.724
PolyMamba-Net (w/o BAM)	0.771	0.708
PolyMamba-Net (Full)	**0.841**	**0.779**

BF1: Boundary F1-score (↑); BIoU: Boundary IoU (↑). Best results are in bold.

## 4 Discussion

### 4.1 Balancing accuracy and efficiency in clinical workflows

The primary objective of this study was to develop a deep learning model capable of accurate polyp segmentation without compromising real-time inference speed. Our results demonstrate that **PolyMamba-Net** effectively bridges the gap between the speed of CNNs and the global contextual understanding of Transformers. While recent Transformer-based models like Swin-UNet (9) have set new benchmarks for accuracy, their quadratic computational complexity ($O\left(N^{2}\right)$) creates a bottleneck for deployment in standard endoscopic towers, which often lack high-end GPUs. By leveraging the linear complexity (O(N)) of the Mamba architecture, our model achieves a throughput of 115 FPS. This is clinically significant because typical video colonoscopy operates at 25–30 FPS. Our model leaves ample computational margin for post-processing or running concurrent algorithms (e.g., optical biopsy classification) without inducing latency, which is a common cause of operator fatigue.

### 4.2 Comparison with recent attention-based methods

Our extended experiments (Table 1) demonstrate that PolyMamba-Net outperforms both ADPNet (11) and AAPCNet (12) across all metrics. ADPNet employs a hybrid CNN-Transformer bridge with an Atrous Self-Attention Pyramid Module and a Dilated Convolution-Transformer Module, achieving a mDice of 0.926 on Kvasir-SEG. While effective, its reliance on attention mechanisms results in 45.8M parameters and 28.6 GFLOPs, compared to our 25.3M parameters and 12.8 GFLOPs—representing a 44.8% reduction in parameters and 55.2% reduction in computation. AAPCNet utilizes asymmetric convolutions and a deep aggregation fusion module based on Res2Net-50, achieving a mDice of 0.921 on Kvasir-SEG with 38.2M parameters. Although it maintains a moderate inference speed (52 FPS), this remains insufficient for dual-task real-time processing pipelines that require concurrent polyp detection and classification.

The superior performance of PolyMamba-Net can be attributed to the inherent advantage of the selective state space mechanism: it captures long-range dependencies without the quadratic cost of self-attention, while the data-dependent gating in Mamba selectively filters relevant visual information based on input content. This makes it more efficient than the explicit attention modules in ADPNet and AAPCNet for capturing the global contextual cues necessary to differentiate polyps from mucosal folds. Furthermore, the cross-dataset evaluation in Table 4 confirms that this advantage is not limited to in-distribution data but extends to unseen polyp distributions, reinforcing the model's robustness.

### 4.3 The role of long-range dependency and boundary refinement

The superior performance of PolyMamba-Net on the Kvasir-SEG dataset (mDice 0.942) can be attributed to its hybrid architectural design. Colonoscopy images often present complex scenarios where polyps are indistinguishable from normal mucosal folds due to similar texture and color. Pure CNNs (e.g., U-Net) struggle in such contexts due to their limited receptive fields. The VSS blocks in our encoder successfully model long-range dependencies, allowing the network to interpret the global geometry of the colon and differentiate true lesions from background noise. Furthermore, the quantitative reduction in Hausdorff Distance (HD95) validates the effectiveness of our Boundary-Aware Module (BAM). In clinical practice, precise boundary delineation is as critical as detection, as it guides the endoscopist in determining the resection margin. Incomplete resection leads to residual neoplastic tissue, a key risk factor for interval cancers (3). The BAM ensures that our model provides sharp, reliable contours rather than the coarse segmentations often produced by baseline methods.

### 4.4 Failure case analysis and robustness

Despite the strong overall performance, we observed several challenging scenarios where PolyMamba-Net produces suboptimal results. First, *diminutive polyps* (< 5 mm) located in deep intestinal folds occasionally yield false negatives, as the polyp occupies only a small fraction of the image and may be occluded by mucosal folds. Second, *flat (sessile) polyps* with extremely low contrast against the surrounding mucosa can result in under-segmentation, particularly when the polyp color closely matches the intestinal wall. Third, images with severe *motion blur* or *specular reflections* (caused by water jets or light sources) degrade segmentation quality, as these artifacts disrupt the feature extraction pipeline. Quantitatively, on Kvasir-SEG images containing specular highlights (approximately 15% of the test set), the mDice drops from 0.942 to approximately 0.891, while for diminutive polyps the mDice decreases to approximately 0.875. These failure modes highlight the need for artifact-aware preprocessing and size-adaptive attention mechanisms in future iterations. Incorporating domain adaptation techniques (21) and multi-scale attention refinement could further improve robustness under these challenging conditions.

### 4.5 Limitations and future work

Despite the promising results, our study has several limitations that warrant discussion.

First, while the cross-dataset evaluation in Section 3.5 demonstrates generalization to unseen polyp datasets, the model has not yet been validated on real-time endoscopic video sequences in a clinical setting. The model processes video frames independently (2D), potentially ignoring the temporal consistency inherent in colonoscopy videos. A polyp visible in frame *t* is likely to exist in frame *t*+1. Future work will explore 3D-Mamba or Recurrent Neural Network (RNN) heads to leverage temporal cues for smoother segmentation, and we plan to conduct a prospective clinical study evaluating the impact of PolyMamba-Net on polyp detection rates during live colonoscopies.

Second, although we utilized public datasets (Kvasir-SEG and CVC-ClinicDB) and performed cross-dataset evaluation on three additional benchmarks (CVC-ColonDB, ETIS-LaribPolypDB, CVC-300), these data may not fully represent the diversity of global populations or rare polyp subtypes (e.g., serrated adenomas). Multi-center external validation on diverse hardware platforms (e.g., edge devices, embedded GPUs in endoscopy towers) is necessary prior to clinical deployment.

Third, current training relied on high-quality still images. Real-world endoscopic feeds often contain motion blur, debris, and water jets. We plan to incorporate domain adaptation techniques to enhance the model's robustness against such artifacts.

Fourth, while we reported statistical significance via the Wilcoxon signed-rank test, future work should include *k*-fold cross-validation on larger multi-center datasets to further strengthen the evidence base.

## 5 Conclusion

In this paper, we proposed PolyMamba-Net, a novel boundary-aware hybrid network for real-time polyp segmentation. By integrating the efficient State Space Model (Mamba) with a convolutional stem, we successfully combined the benefits of global context modeling and local feature extraction. Extensive experiments demonstrate that PolyMamba-Net outperforms state-of-the-art methods—including recent attention-based architectures such as ADPNet and AAPCNet—in segmentation accuracy, with all improvements confirmed as statistically significant (*p* < 0.05). Furthermore, cross-dataset evaluations on three unseen benchmarks validate the model's generalizability. Our model achieves inference speeds three times faster (115 FPS) with only 25.3M parameters and 12.8 GFLOPs, making it the most efficient high-accuracy method evaluated. These findings suggest that Mamba-based architectures represent a viable and superior alternative to Transformers for medical image analysis, particularly in resource-constrained real-time environments. Our work provides a strong foundation for the development of next-generation “Smart Endoscopy” systems, potentially reducing the rate of missed diagnoses and improving patient outcomes in colorectal cancer screening.

## Data Availability

The original contributions presented in the study are included in the article/supplementary material, further inquiries can be directed to the corresponding author.
